# Proteomic analysis of differential responses to norflurazon herbicide in the model green alga *Chlamydomonas reinhardtii*

**DOI:** 10.1038/s41598-025-17119-6

**Published:** 2025-08-27

**Authors:** Kantinan Leetanasaksakul, Thanaporn Intha, Sittiruk Roytrakul, Narumon Phaonakrop, Siriwan Thaisakun, Anchalee Sirikhachornkit

**Affiliations:** 1 National Center for Genetic Engineering and Biotechnology, National Science and Technology Development Agency, 113 Paholyothin Road, Klong 1, Klong Luang, Pathumthani, 12120 Thailand; 2 Department of Genetics, Faculty of Science, Kasetsart University, Bangkok, 10900 Thailand

**Keywords:** Carotenoid, *Chlamydomonas*, Herbicide, Norflurazon, Proteomic, Biotechnology, Genetics, Microbiology, Plant sciences

## Abstract

Norflurazon is a widely utilized pesticide in agriculture for weed management. The mechanism of action involves the inhibition of an initial step in carotenoid synthesis. This inhibition results in the instability of the photosynthetic machinery and subsequent cell bleaching. Microalgae have attracted significant interest for the production of valuable products. Nonetheless, the mass cultivation of microalgae continues to encounter many challenges that result in high production costs. A potential issue in photobioreactor and open pond cultivation is contamination by other microalgae, which can destroy the mass culture entirely. Strains exhibiting greater resistance to specific chemicals may be beneficial in reducing contamination from other algae. Furthermore, integrating microalgal production with phycoremediation constitutes a sustainable approach to the circular economy. Many norflurazon-resistant microalgae strains have been developed, including the model unicellular green microalga *Chlamydomonas reinhardtii*. In previous studies, mutant and transgenic strains resistant to high concentrations of norflurazon have been generated to study herbicide tolerance in *Chlamydomonas reinhardtii*. Nonetheless, the application of genetically engineered organisms should remain cautious. Moreover, mutant strains generated through conventional methods were created using very high chemical concentrations. The effects of introducing such strains on the composition of organisms in the environment remain a concern. This study investigated the feasibility of utilizing natural isolates of this alga for mass production in the presence of norflurazon. Twenty isolates of this alga were evaluated for tolerance to norflurazon. The two most tolerant isolates demonstrated the ability to withstand 5–10 µM of norflurazon, a concentration previously employed to select mutants and transformants resistant to norflurazon. Physiological and proteomic data revealed an enhancement of photosynthesis and photoprotection processes as the primary mechanism for norflurazon tolerance in one isolate, whereas another isolate demonstrated a reduction in protein synthesis, photosynthesis, and cell motility.

## Introduction

Agricultural herbicides are extensively utilized and their application is rising in response to the growing global population and the demand for increased food production. Norflurazon is a herbicide commonly employed in agricultural weed management. It is approved for use in soil for cotton, soybean, tree fruits, nut crops, citrus, and cranberries^1^. Norflurazon interacts with the enzyme phytoene desaturase, catalyzing the conversion of phytoene into zeta-carotene, an early step in carotenoid biosynthesis^2,3^. This inhibition results in chloroplast bleaching, photooxidation, and chlorophyll depletion, which subsequently inactivates the photosynthetic apparatus, causes photobleaching of green tissues, and ultimately leads to cell death^4^. Norflurazon has been documented to leach into soil and aquatic environments. It has been detected in groundwater monitoring studies and is present in deep soil layers, surface water, and underground water^5^. The substance exhibits a half-life ranging from approximately 38 to 731 days, contingent upon soil characteristics. However, on the soil surface, photodegradation leads to a half-life of roughly 41 days^6,7^. The toxicity level of norflurazon is labeled as very toxic to aquatic life^8,9^. The European Chemical Agency (ECHA) has classified it as highly toxic to aquatic organisms, exhibiting long-lasting effects^9^. Norflurazon is classified as a possible human carcinogen^6^. Effective management of agricultural waste contaminated by norflurazon is crucial to reduce negative impacts on human and animal health.

Microalgae are small photosynthetic organisms that are found in various ecosystems. Eukaryotic microalgae are capable of converting carbon dioxide and light energy into valuable compounds such as proteins, pigments, and lipids. Over the past few decades, there have been efforts to utilize microalgae for bioenergy and biofuels, including biodiesel, biogas, biohydrogen, food, feed, and pharmaceuticals^10,11^. Interest in the mass cultivation of microalgae has increased, utilizing both closed systems via photobioreactors (PBRs) and open systems through open raceway ponds^12^. Such Large-scale commercial production of microalgae is acknowledged as a renewable and environmentally sustainable method for feedstock production, complementing small-scale traditional cultivations aimed primarily at human or animal consumption^10^. Consequently, cultivating microalgae presents a feasible method for generating biomass applicable in various industrial sectors, including the production of bioactive compounds, recombinant proteins, animal feed, biofuels, organic fertilizers, and biostimulants^13–17^. However, numerous compounds have yet to be industrially produced using microalgae. Large-scale cultivation encounters numerous obstacles that increase costs^18,19^. Maintaining axenic or near-axenic algal cultures is crucial, as contaminating organisms reduce the production capacity of algal bioreactors or raceways. In addition to contamination, competition from other microalgal strains poses a significant challenge, particularly in open-pond production systems. The cost of production presents a significant challenge during various stages, such as algal cell collection, dewatering, and the extraction process. One strategy to mitigate contamination and competition from other microalgae involves utilizing a strain that exhibits resistance to specific chemicals such as herbicides in agricultural wastewater. Microalgae cultivation in wastewater on marginal soils also mitigates competition with food crops for water and arable land^20,21^.

Hence, the use of agricultural wastewater contaminated with herbicides like norflurazon for large-scale algal cultivation offers a sustainable and cost-effective source of nutrients, representing a feasible approach for a circular economy. Numerous studies have documented the generation of diverse norflurazon-resistant microalgal strains through both forward and reverse genetic methods.

Mutant and transgenic strains resistant to norflurazon, which inhibit carotenoid synthesis, have been reported to accumulate elevated carotenoid levels. Modification of specific bases in the *phytoene desaturase* (*pds*) gene, which confers resistance to norflurazon, has been proposed as a selectable marker in microalgae^22,23^. The acceptance of genetically modified organisms has increased in recent years, especially in developing countries; nonetheless, discussions continue concerning social and ethical issues. Challenges include biosafety concerns, particularly the uncertain long-term effects of genetically modified products on ecosystems and human health^24–26^as well as risks associated with the use of antibiotic resistance marker genes. Key ecological concerns involve the impact of transgenic algae on gene flow, biodiversity, and ecosystems, especially in open cultivation settings. Even though random mutagenesis and adaptive laboratory evolution produce mutations in the absence of transgenes, the capacity to withstand specifically high concentrations of xenobiotics warrants attention. These strains were not a product of natural occurrence but were instead chosen under unusual conditions. The influence of these organisms on the composition of other organisms in nature remains unclear.

The model green unicellular microalga *Chlamydomonas reinhardtii* has been utilized to genetically modify the *pds* gene and to identify norflurazon-resistant strains^22,23,27^. This model alga possesses rapid growth, genetic manipulability, and extensive research and omics data accumulated over several decades. This has functioned as a paradigm for research on biodiesel and other high-value products^28^. Recent advancements in the scale-up of *C. reinhardtii* indicate that this species may soon become a viable industrial producer^29,30^. This study evaluated the tolerance of twenty isolates of *C. reinhardtii* to different concentrations of norflurazon. The physiological and proteomic responses of three isolates exhibiting differing tolerance levels were analyzed and discussed. Our findings indicated that wild-type isolates of *C. reinhardtii* exhibiting varied tolerance to herbicides could be utilized for the mass production of this alga.

## Materials and methods

### Algal isolates and culture conditions

A total of 20 isolates of *Chlamydomonas reinhardtii* were used in this study. These isolates included S1D2, CC-125, CC-407, CC-408, CC-1009, CC-1010, CC-1373, CC-1690, CC-1952, CC-2342, CC-2343, CC-2344, CC-2931, CC-2932, CC-2935, CC-2936, CC-2937, CC-2938 and CC-4414. All isolates were obtained from the Chlamydomonas Resource Center at the University of Minnesota, with the exception of 4 A+, which was graciously provided by Prof. Krishna Niyogi (University of California, Berkeley). Cells were maintained on TAP agar plates at 25 °C.

To investigate the impact of norflurazon on growth of *Chlamydomonas* on TAP agar plates, a 3 µL aliquot from two dilutions (1 × 10^6^ cell mL^− 1^ and 1 × 10^5^ cell mL^− 1^) were spotted onto plates containing norflurazon concentrations (Sigma-Aldrich, Poole, UK). These plates were then incubated at 25 °C under an illumination of 200 µmoles photons m^− 2^s^− 1^ for one week. Liquid cultures were prepared using 50 mL of TAP medium with 0 µM, 1 µM, 5 µM, and 10 µM of norflurazon. These liquid cultures were incubated at 25 °C, with continuous shaking at 120 rpm, and exposed to an illumination intensity of 200 µmoles photons m^− 2^s^− 1^. Before conducting experiments, cultures in the logarithmic growth phase were diluted to a density of 1 × 10^6^ cell mL^− 1^. All experiments were conducted with three biological replicates to ensure the reliability of the results.

### Growth and cell morphology

Standard curves depicting the correlation between cell density and optical density at 750 nm were constructed by counting cells using a hemocytometer. To determine cell density, 1 mL of culture was used to measure the optical density at 750 nm. The morphology of the cells was assessed using a light microscope.

### Cell viability assay

Cell viability was determined using the Evans blue staining method^31^. This technique relies on measuring the proportion of cells that did not take up Evans blue dye, a dye that selectively stains nonviable cells, to estimate cell viability. A 1 mL volume of cells was exposed to a 1% (w/v) Evans blue solution for 15 min. Unbound dye, which remained associated with viable cells, was subsequently removed through thorough washing with fresh TAP medium. Dye bound to deceased cells was solubilized using a solution consisting of 50% (v/v) methanol and 1% (w/v) sodium dodecyl sulfate (SDS) for 30 min at 50 °C, and its concentration was assessed by measuring absorbance at 600 nm. The data is presented as a percentage of overall cell mortality, standardized against Evans blue staining of equivalent cells that had been subjected to heat treatment to complete mortality (approximately 100% mortality)^32,33^.

### Photosynthetic activity and pigment content

The measurement of the fluorescence parameter assessing the quantum yield of PSII (F_v_/F_m_) was conducted using Z985 Cuvette AquaPen (Qubit Systems, Ontario, Canada). Before measurement, a culture volume of 1 mL was kept in the dark for at least 20 min. To estimate the contents of chlorophylls and carotenoids, pigment extraction was carried out on 1 mL of the culture. Cells were harvested by centrifugation at 7500 × g for 5 min and then resuspended in 1 mL of 80% acetone. Extraction was facilitated through vortexing until the cells became colorless. Following another round of centrifugation, the supernatant was analyzed using a spectrophotometer, measuring optical densities at 470, 646, and 663 nm. The pigment concentrations were calculated using established Eq.^34^. To ensure accuracy in all measurements, an absorbance reading at 720 nm was employed for correction to account for any potential contamination from colored compounds^35^.

### Protein extraction

The methods of protein extraction and digestion follow the procedures previously described^36^. In brief, cells collected after 48 h of each treatment were ground into fine powder in a mortar with liquid nitrogen. For each sample, 200 mg of wet cell weight was used to ensure equal biomass input for all downstream analyses, regardless of total biomass produced. Cells were extracted with a solution of 0.5% sodium dodecyl sulfate (SDS) (w/v), with continuous vortexing for a duration of 3 h at room temperature. Subsequently, the samples were subjected to centrifugation at 8000 rpm for 10 min at room temperature. The supernatant was then carefully transferred to a new microcentrifuge tube and combined with 72% trichloroacetic acid (TCA) (w/v) and 0.15% deoxycholate (w/v). These mixtures were incubated overnight at − 20 °C to ensure complete precipitation. Subsequently, the mixtures were centrifuged at 10,000 rpm for 10 min at room temperature. The pellets were washed using cold acetone until they appeared white. The resuspension of the pellets was diluted in a 0.5% SDS solution, and the protein concentration was determined using the Lowry method^37^.

### Protein digestion

The samples underwent an in-gel digestion process. They were first subjected to treatment with 10 mM dithiothreitol (DTT) in 10 mM ammonium bicarbonate (AMBIC) to reduce disulfide bonds. This treatment took place at 60 °C for 1 h. Following reduction, the sulfhydryl groups were alkylated by adding 15 mM iodoacetamide in 10 mM AMBIC at room temperature. This alkylation step was carried out in the dark for 45 min. Subsequently, the protein samples were digested using 50 ng/µL of sequencing-grade trypsin (1:20 ratio) (Promega, Madison, WI, USA). The digestion process was performed at 30 °C over a 16-h duration. Finally, the resulting peptides were dried using a speed vacuum concentrator and protonated with 0.1% formic acid (v/v) before being subjected to LC-MS/MS analysis.

### Liquid chromatography-tandem mass spectrometry (LC-MS/MS)

The tryptic peptides were prepared for injection into a Ultimate3000 Nano/Capillary LC System (Thermo Scientific, UK), coupled to a SCIEX Zeno TOF 7600 system (Darmstadt, Germany) with a TwinSpray Turbo V ion source. Positive electrospray ionization was employed. Peptides were initially enriched using a 300 μm inner diameter, 5 mm C18 Pepmap 100 column (5 μm particle size, 100 Å pore size) (Thermo Scientific, UK). Subsequently, separation occurred on a 75 μm inner diameter, 15 cm column packed with 2 µmAcclaim PepMap RSLC C18 material (100 Å pore size) via the nanoViper system (Thermo Scientific, UK). Solvent A (0.1% formic acid in water) and Solvent B (0.1% formic acid in 80% acetonitrile) were used for column supply. A gradient of 5–55% Solvent B was applied at a constant flow rate of 0.30 µl/min over 30 min. Electrospray ionization operated at 3.3 kV, and positive-ion mode MS and MS/MS spectra were acquired in the m/z range of 350–1800 using SCIEX OS Software 3.1 (https://sciex.com/products/software/sciex-os-software).

### Protein identification

The LC-MS/MS data were processed using MaxQuant version 2.1.4.0 (Max-Planck Institute for Biochemistry, Martinsried, Germany) for peptide MS signal intensities. This analysis employed the built-in Andromeda search engine for label-free quantification^38^. A search against the Uniprot database, dated August 3, 2023, was performed for protein identification, with specific criteria including taxonomy (Chlamydomonas sp.), enzyme (trypsin), variable modifications (carbamidomethyl, oxidation of methionine residues), monoisotopic mass values, unrestricted protein mass, peptide mass tolerance (1.2 Da), fragment mass tolerance (± 0.6 Da), peptide charge states (1+, 2+, and 3+), and maximum allowed missed cleavages.

Results were documented in a file named “proteinGroup.txt”. Subsequent data processing, following the methodology previously described^39^aimed to detect variations in protein levels between the treated with norflurazon condition and the control group. This involved assessing fold-changes and associated p-values based on protein abundance calculations. To summarize, abundance data underwent two forms of normalization after being converted to logarithmic values. The first normalization method scaled each value relative to the average of all protein abundances within the specific sample. The second normalization method adjusted values based on their distribution width to account for potential differences in acquisition dynamic range across samples. To address missing values, a probabilistic minimum imputation model was applied, assuming resampling for low-abundance data. Low abundance was defined as values more than 2 standard deviations smaller than the distribution of valid values, with a resampling variability set at 0.3. Fold-changes were computed as the ratio of transformed and normalized protein abundance values between each sample and the control group. P-values were determined through various t-tests, depending on whether replicates for each protein exhibited homoscedastic or heteroscedastic behavior, as determined by F-tests.

Gene ontology (GO) was identified using Uniprot (http://www.uniprot.org) and associated search tools. For visualization, overlapping proteins were generated using Jvenn^40^. Noteworthy protein expression levels were visualized using a volcano plot^41^. Principal Component Analysis with Linear Discriminant Analysis (PLS-DA) was conducted through the MetaboAnalyst web-based platform^42^.

### Statistical analysis

Statistical analysis was performed using SPSS Statistics version 22.0, and significance was determined using analysis of variance (ANOVA) with a threshold of *p* < 0.05. All experiments were conducted with three biological replicates, and the results are presented as mean values ± standard deviation^43^.

### Data availability

Mass spectrometry proteomics data are available via ProteomeXchange: PXD057458 (https://proteomecentral.proteomexchange.org/cgi/GetDataset?ID=PXD057458 ) and JPOST: JPST003453 (https://repository.jpostdb.org/entry/JPST003453).

## Results

### Effect of Norflurazon on cell growth

**Fig. 1:**
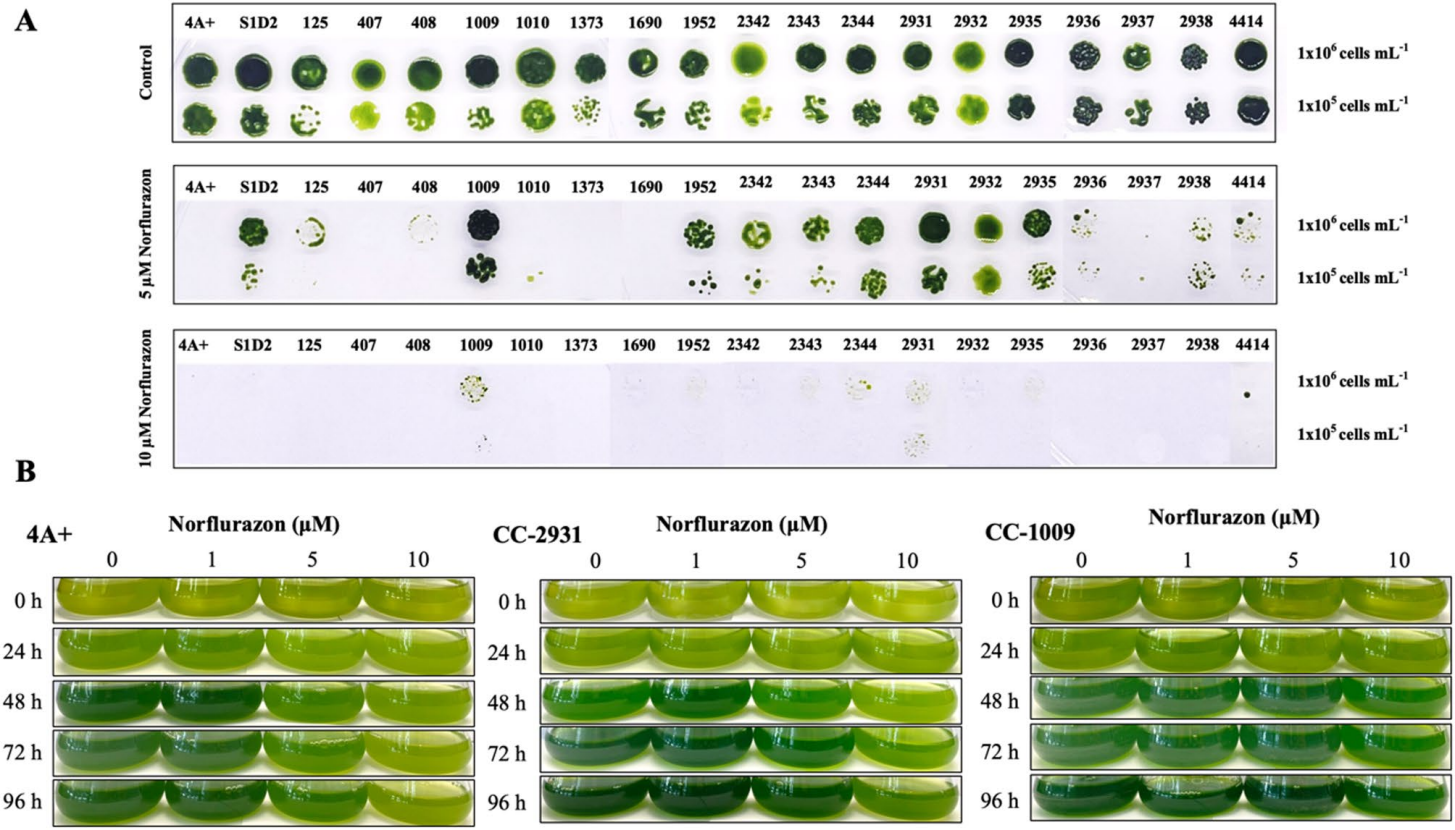
Growth of 20 *C. reinhardtii* isolates including 4 A+, S1D1, CC-125, CC-407, CC-408, CC-1009, CC-1010, CC-1373, CC-1690, CC-1952, CC-2342, CC-2342, CC-2344, CC-2931, CC-2932, CC-2935, CC-2936, CC-2937, CC-2938, and CC-4414 cells on plates with and without norflurazon (**A**). Cultures were diluted to indicated density (right) and three microliters were spotted onto plates. Norflurazon concentration was shown on the left. The isolate number is shown on top of each condition. Growth in liquid culture of 4 A+, CC-2931, and CC-1009, under different concentrations of norflurazon (**B**). Cells were diluted to a density of 1 × 10^6^ cells mL^− 1^, Norflurazon was added at the indicated concentration. Photographs were taken every day for 4 days.

Twenty *C. reinhardtii* isolates were screened to determine their differential responses to norflurazon. Their growth was observed on plates with 0, 5, and 10 µM norflurazon. All isolates exhibited comparable growth on plates devoid of norflurazon; however, variations in cell growth were seen at 5 and 10 µM norflurazon (Fig. 1A). At 5 µM, nine isolates exhibited significant growth, whereas others had minimal or no growth development. CC-1009 and CC-2931 exhibited superior growth compared to the others. Both isolates exhibited observable growth at 10 µM norflurazon. Consequently, they were chosen for additional studies with 4 A+, which exhibited sensitivity to norflurazon. The three isolates were cultivated in liquid medium with 0, 1, 5, and 10 µM norflurazon (Fig. 1B). The presence of norflurazon resulted in a lighter appearance of the cells, and this impact is proportional to the concentration. The sensitive strain, 4 A+, exhibited the most significant bleaching, whereas the most resistant isolate, CC-1009, preserved a dark green phenotype at both 5 and 10 µM norflurazon.

**Fig. 2:**
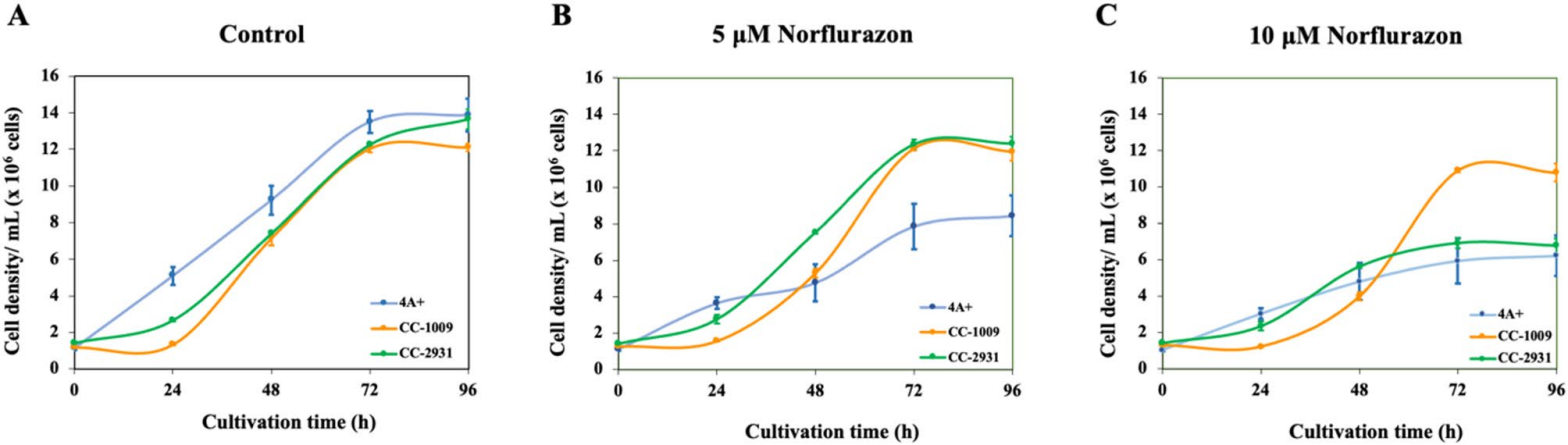
Cell density under different norflurazon concentrations. Blue, orange, and green lines are 4 A+, CC-1009, and CC-2931, respectively. All data represent the mean ± SD of three biological replicates. Asterisks (*) indicate significant differences between the control and norflurazon within the same condition (*p* < 0.05).

In the absence of norflurazon, all isolates exhibited fast development during the initial 72 h, followed by a stabilized cell density from 72 to 96 h (Fig. 2). The introduction of norflurazon resulted in a dose-dependent decrease in growth for all isolates. 4 A + exhibited the highest cell density across all three isolates under all conditions during the initial 24 h (Fig. 2A–C). In the presence of norflurazon, the cell density of 4 A + decreased relative to other strains at subsequent periods (Fig. 2B,C). 4 A + demonstrated a notable reduction in cell density at 96 h, attaining 39% and 55% at 5 and 10 µM norflurazon, respectively. In a comparison of the norflurazon-resistant isolates CC-1009 and CC-2931 at 5 µM norflurazon, CC-2931 initially demonstrated a greater cell density than CC-1009; however, the densities converged at subsequent time points (Fig. 2B). At 10 µM norflurazon, CC-2931 exhibited increased cell density during the initial phase of the experiment; however, this density significantly declined to match that of 4 A + in the latter phase, while CC-1009 successfully sustained a high cell density (Fig. 2C).

**Fig. 3:**
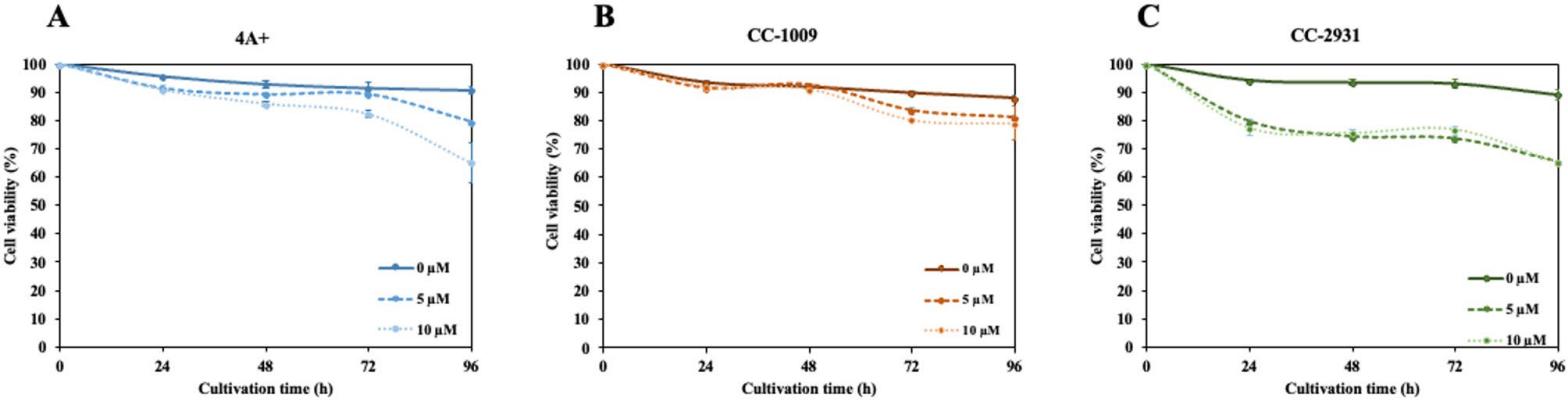
Cell viability under norflurazon. Full lines, dashed lines, and dotted lines represent cultures with 0 µM, 5 µM, and 10 µM norflurazon, respectively. All data are means ± SD of three biological replicates. Significant differences between the control culture and chemical-added culture within the same condition are indicated by asterisks (*) (*p* < 0.05).

Evans blue staining was conducted to evaluate cell viability. Norflurazon at concentrations of 5 µM and 10 µM markedly decreased cell viability in 4 A + and CC-2931 (Fig. 3A,C). CC-2931 exhibited the lowest cell viability % among the three isolates, with effects observable as early as 24 h. CC-1009 subjected to norflurazon treatments exhibited comparable cell viability to the control group without norflurazon during the initial 48 h (Fig. 3B). Following a 48-hour period, cells exposed to norflurazon exhibited reduced vitality compared to the control group. Nonetheless, the cell viability of these cells was the highest among all three isolates.

### Photosynthesis and pigment content

**Fig. 4:**
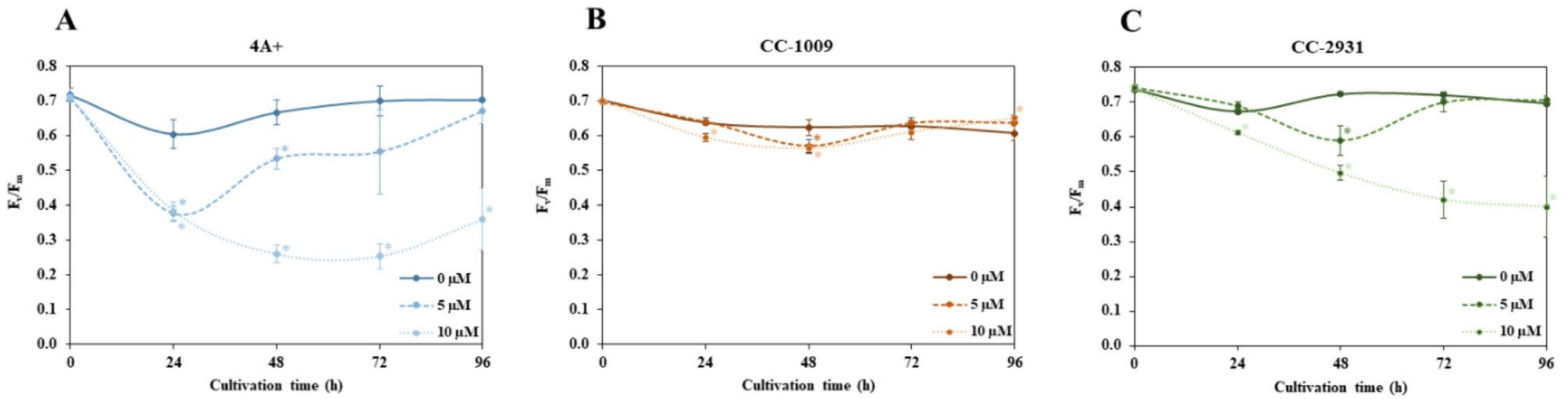
Maximum quantum yield of PSII (Fv/Fm) in the presence of norflurazon in 4 A+ (**A**), CC-1009 (**B**), and CC-2931 (**C**). Full lines, dashed lines, and dotted lines represent cultures with 0 µM, 5 µM, and 10 µM norflurazon, respectively. The data presented are the mean ± SD of three biological replicates. Any significant distinctions between the control culture and the culture with added norflurazon under the same conditions are denoted by asterisks (*) (*p* < 0.05).

Efficient light utilization is crucial for optimizing algal development, particularly in outdoor settings with fluctuating light intensity. F_v_/F_m_, an indicator of photosynthetic efficiency, serves to evaluate algal health by assessing light utilization efficiency in photosystem II (PSII). Under control conditions (0 µM norflurazon), F_v_/F_m_ values for all isolates remained consistent, ranging from 0.60 to 0.78 (Fig. 4A–C). 4A + had a significant decline as early as 24 h, as seen by a marked reduction in this value (Fig. 4A). Notably, at the final time point, the value returned to normal at 5 µM, however at 10 µM, it remained significantly diminished. In CC-1009, the administration of norflurazon exhibited no significant impact on photosynthetic efficiency (Fig. 4B). For CC-2931, the concentration at 5 µM exhibited minimal impact on this value. At 10 µM, the F_v_/F_m_ values consistently decline over time.

**Fig. 5:**
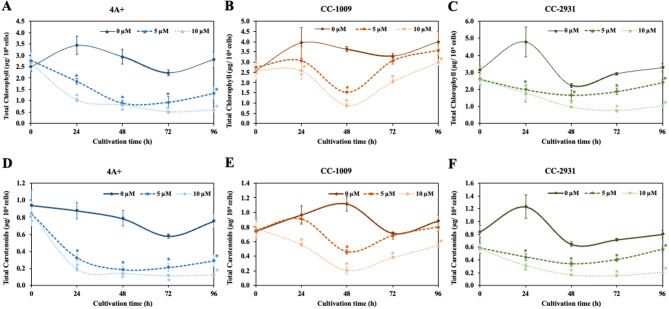
Chlorophyll content (**A**–**C**) and carotenoid content (**D**–**F**) in the presence of norflurazon. Full lines, dashed lines, and dotted lines represent cultures with 0 µM, 5 µM, and 10 µM norflurazon, respectively. The data presented are the mean ± SD of three biological replicates. Significant differences between the control culture and the culture treated with norflurazon under identical conditions are indicated by asterisks (*) (*p* < 0.05).

In the absence of norflurazon, pigment concentration, including chlorophylls and carotenoids, was comparable between 4 A + and CC-2931. CC-1009 exhibited a comparable concentration of carotenoids, although demonstrated an elevated chlorophyll content (Fig. 5A–F). Norflurazon significantly decreased the cellular levels of these pigments (Fig. 5A–E). Notably, at the 96-hour mark, CC-1009 exhibited a recovery, demonstrating elevated levels of both pigments in comparison to 4 A + and CC-2931 (Fig. 5A–E).

**Fig. 6:**
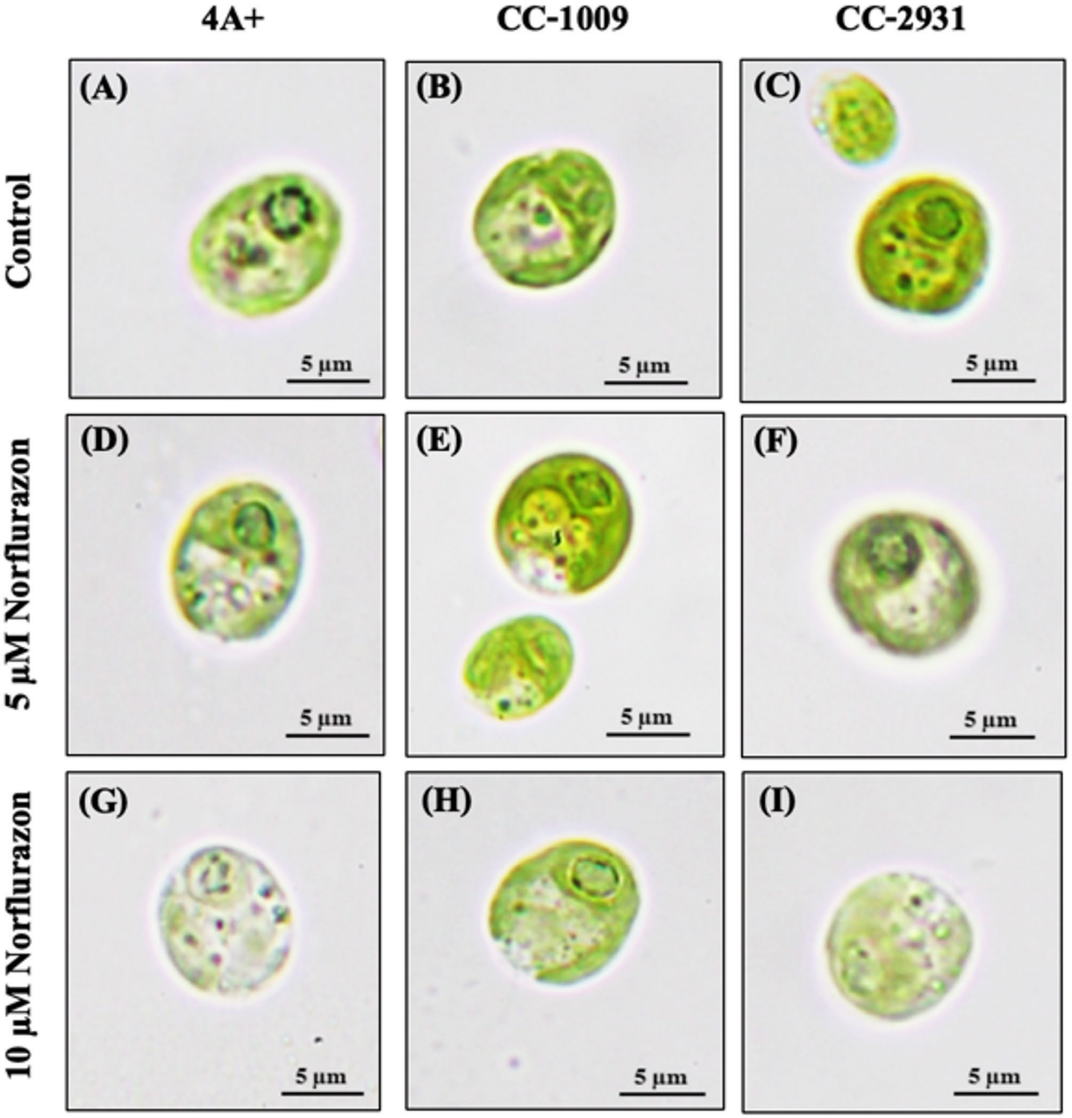
Morphological alterations following 96 h of norflurazon exposure. Cells were cultured in TAP medium (**A**–**C**) and TAP medium containing norflurazon (**D**–**I**).

The impact of norflurazon on changes in cell shape and pigmentation was examined. In the absence of norflurazon, all isolates demonstrated ellipsoidal or nearly spherical cells, approximately 10 μm in diameter, exhibiting a dense internal distribution of chlorophyll pigment (Fig. 6). However, in the presence of norflurazon, a decrease in chlorophyll content was noted with rising concentrations (Fig. 6A–C). A significant bleaching effect on chlorophyll pigments was detected at 10 µM norflurazon. As a result, 4 A + underwent considerable chlorophyll pigment bleaching, followed by CC-2931 and CC-1009 (Fig. 6G–I).

**Fig. 7:**
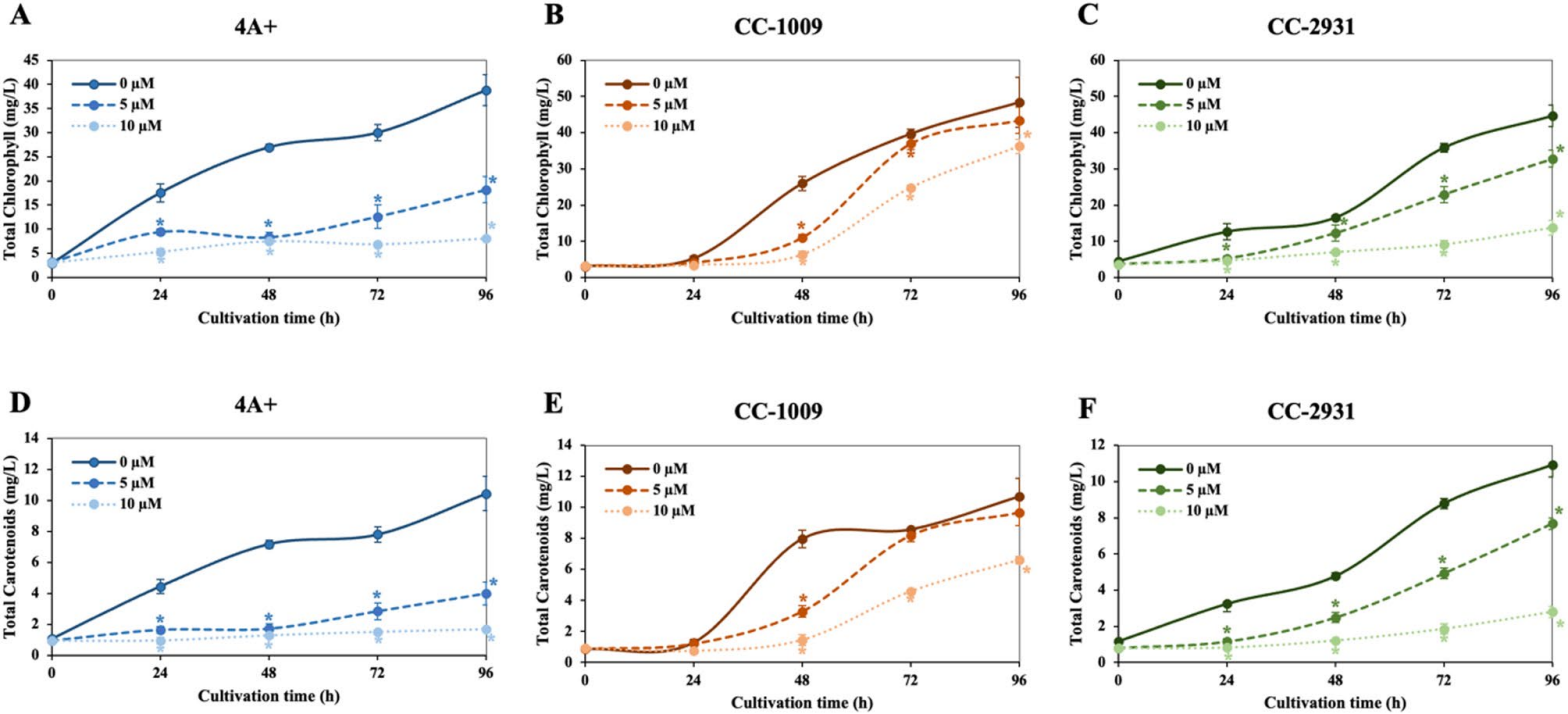
Chlorophyll production (**A**–**C**) and carotenoid production (**D**–**F**). Full lines, dashed lines, and dotted lines represent cultures with 0 µM, 5 µM, and 10 µM norflurazon, respectively. All data were means ± SD from three biological replicates. Significant variations between the control culture and the chemical-treated culture under the same conditions are indicated by asterisks (*) (*p* < 0.05).

Microalgae have been proposed for the commercial production of many high-value chemicals, including lipids and pigments. To assess alterations in the production of pigments induced by norflurazon, chlorophyll and carotenoid yields per unit volume of culture were determined. Norflurazon decreased the production of both pigments in a dose-dependent fashion, exhibiting the most pronounced effect on 4 A+, followed by CC-2931 and CC-1009 (Fig. 7A–F). The decrease in pigment production in 4 A + and CC-2931 began very early at 24 h, whereas CC-1009 showed a reduction in pigment production only after 48 h (Fig. 7A–C). At 5 µM norflurazon, the production of both pigments in CC-2931 was diminished; however, in CC-1009, the production of both pigments was able to return to the control level without norflurazon (Fig. 7B,C,E,F).

### Proteomic analysis

**Fig. 8:**
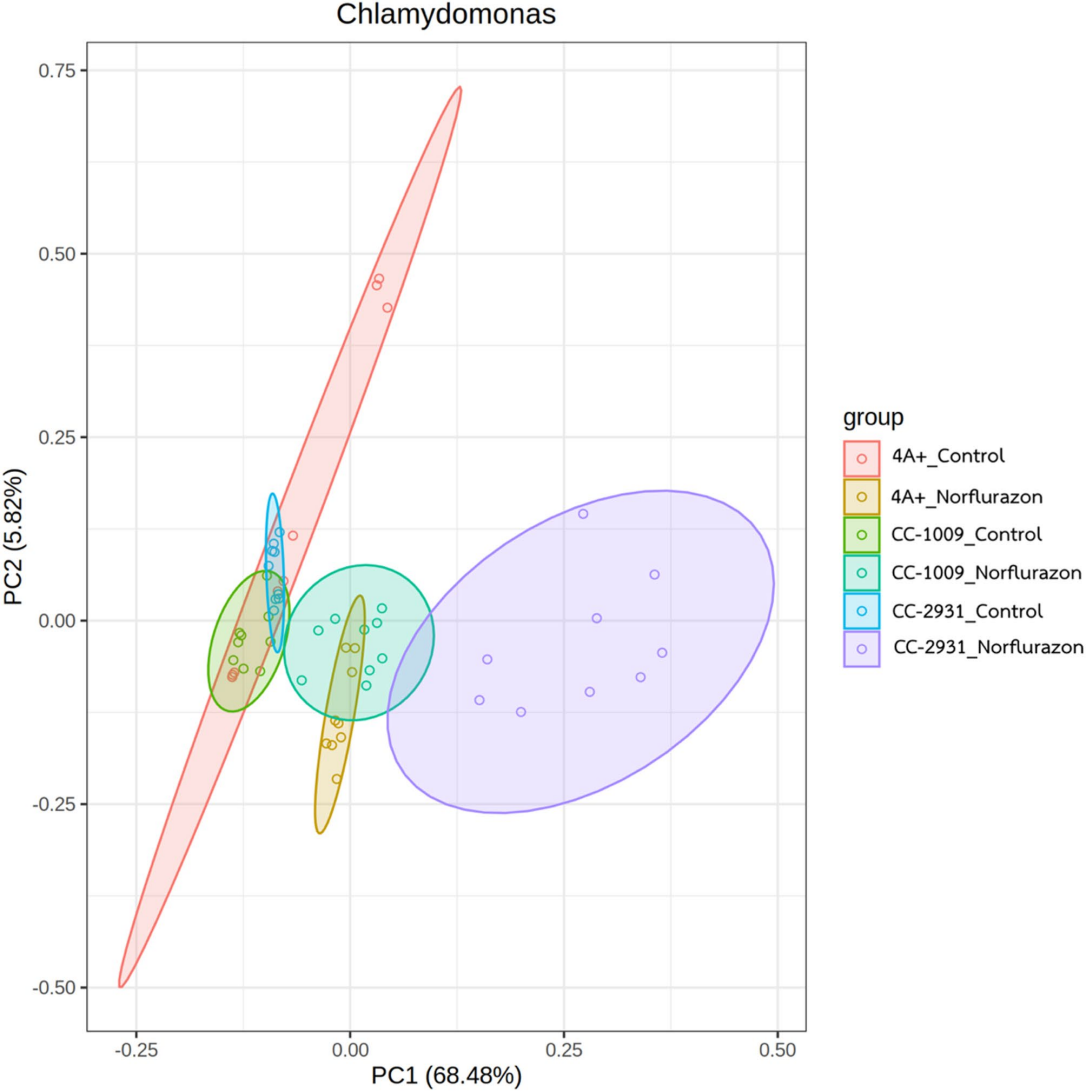
Principal Component Analysis (PCA) reveals distinct protein expression patterns in *Chlamydomonas* samples between control conditions (absence of norflurazon) and norflurazon treatment. The analysis distinguishes three primary clusters through PC1 (45.2%) and PC2 (18.6%, jointly explaining 63.8% of the observed variance. Sample groups demonstrate distinct spatial distribution: Control samples from all isolates cluster together, showing clear separation from norflurazon-treated samples which aggregate towards the right quadrant of the PCA plot. The observed clustering patterns reflect robust biological replication and distinct protein expression signatures across experimental conditions. With minimal intra-group variation and clear inter-group seperation along both principal component axes.

The Principal Component Analysis of *Chlamydomonas* protein expression data demonstrates striking separation between norflurazon-treated and control samples across all three isolates. The two-dimensional PCA plot reveals that norflurazon treatment induces substantial changes in protein expression patterns, creating distinct spatial clustering between treated and untreated conditions (Fig. 8).

Control samples from all three *Chlamydomonas* isolates aggregate primarily in the left quadrant, with each isolate maintaining its characteristic clustering pattern that reflects distinct baseline protein profiles. Conversely, upon norflurazon treatment, samples demonstrate a pronounced shift toward the right quadrant, indicating a consistent and substantial reorganization of protein expression patterns in response to the treatment. This clear spatial separation between control and treated samples along the primary component axis emphasizes the significant impact of norflurazon on the cellular proteome.

This protein expression landscape reveals that norflurazon treatment induces substantial and consistent changes in the *Chlamydomonas* proteome, while preserving isolate-specific characteristics. The clear spatial separation between treated and untreated samples across all isolates underscores the profound impact of norflurazon on cellular protein expression patterns.

**Fig. 9:**
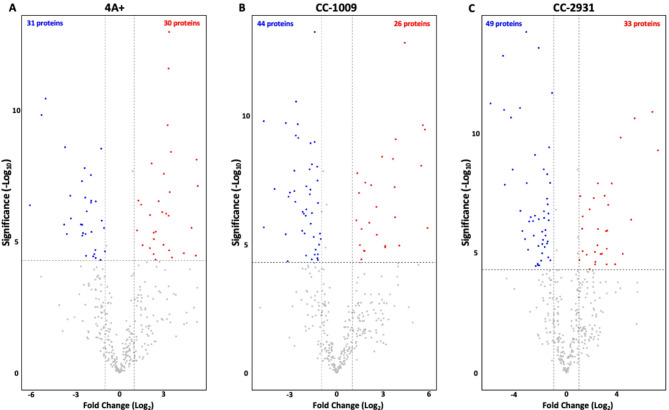
Summary of differentially expressed proteins (**A**–**C**). Volcano plots were employed to analyze the differentially expressed proteins in 4 A+, CC-1009, and CC-2931. These plots illustrate all the detected proteins as gray dots across all samples, following the filtering and normalization steps. The x-axis represents the log2 fold-change in protein abundance between untreated and 10 µM norflurazon-treated conditions, while the y-axis indicates the -log10 value of the corresponding p-value, denoting statistical significance. Each dot on the plot corresponds to an individual protein. Proteins with a log2 fold-change value less than − 1 or greater than 1 and a − log10(p-value) greater than 4.32 (equivalent to a raw p-value less than 0.05) are highlighted and color-coded to indicate their significance. Up-regulated proteins are shown in red, while down-regulated proteins are shown in blue.

An overview of the fold change, highlighting the differentially expressed proteins between each group pair, was established using the criteria (log2|fold-change| ≥ 1 and *p* < 0.05). The results indicated that norflurazon treatment resulted in the upregulation of 30 proteins and the downregulation of 31 proteins in 4 A+ (Fig. 9A). In CC-1009, twenty-six proteins demonstrated upregulation, whereas forty-five proteins indicated downregulation (Fig. 9B). In the case of CC-2931, thirty-three proteins exhibited upregulation, whereas forty-nine proteins showed downregulation (Fig. 9C).

**Fig. 10:**
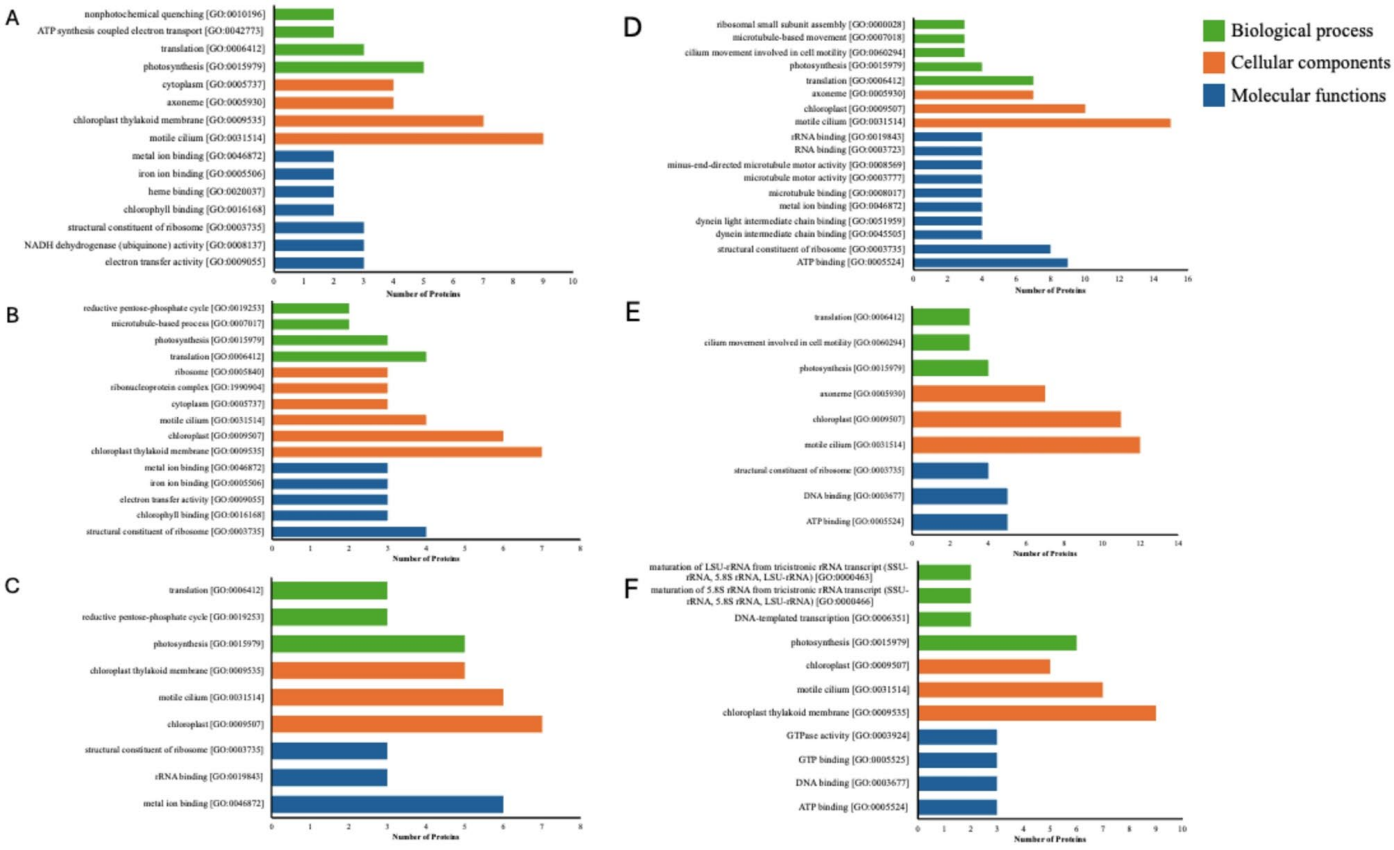
Gene ontology (GO) analysis of differential expression in up-regulated proteins for CC-2931 (**A**), CC-1009 (**B**), and 4 A+ (**C**), as well as down-regulated proteins in CC-2931 (**D**), CC-1009 (**E**), and 4 A+ (**F**). The categories include biological processes (green), cellular components (brown), and molecular functions (blue).

The upregulated proteins were classified via gene ontology (GO) analysis for biological processes, cellular components, and molecular functions. The primary biological activities associated with CC-2931 were photosynthesis (GO:00015979), translation (GO:0006412), ATP synthesis coupled with electron transport (GO:0042773), and nonphotochemical quenching (GO:0010196) (Fig. 10A). In CC-1009, the pathways of translation (GO:0006412), photosynthesis (GO:0015979), microtubule-based processes (GO:0007017), and the reductive pentose-phosphate cycle (GO:0019253) were the majority (Fig. 10B). For 4 A+, the categories included photosynthesis (GO:0015979), the reductive pentose-phosphate cycle (GO:0019253), and translation (GO:0006412) (Fig. 10C).

CC-2931 demonstrated the presence of motile cilium (GO:0031514), chloroplast thylakoid membrane (GO:0009535), axoneme (GO:0005930), and cytoplasm (GO:0005737) as its principal cellular components (Fig. 10A). For CC-1009, the following components were included: chloroplast thylakoid membrane (GO:0009535), chloroplast (GO:0009507), motile cilium (GO:0031514), cytoplasm (GO:0005737), ribonucleoprotein complex (GO:1990904), and ribosome (GO:0005840) (Fig. 10B). For 4 A+, the identified components were chloroplast (GO:0009507), motile cilium (GO:0031514), and chloroplast thylakoid membrane (GO:0009535) (Fig. 10C).

Regarding molecular functions, CC-2931 exhibited electron transfer activity (GO:0009055), NADH dehydrogenase (ubiquinone) activity (GO:0008137), structural constituent of ribosome (GO:0003735), chlorophyll binding (GO:0016168), heme binding (GO:0020037), iron ion binding (GO:0005506), and metal ion binding (GO:0046872) (Fig. 10A). CC-1009 demonstrated structural constituents of ribosome (GO:0003735), chlorophyll binding (GO:0016168), electron transfer activity (GO:0009055), iron ion binding (GO:0005506), and metal ion binding (GO:004672) (Fig. 10B). For 4 A+, the functions included metal ion binding (GO:0046872), rRNA binding (GO:0019843), and structural constituent of ribosome (GO:0003735) (Fig. 10C).

By contrast, the biological processes associated with down-regulated protein in CC-2931 included translation (GO:0006412), photosynthesis (GO:0015979), cilium movement involved in cell motility (GO:0060294), microtubule-based movement (GO:0007018), and ribosomal small subunit assembly (GO:0000028) (Fig. 10D). In CC-1009, they were photosynthesis (GO:0015979), cilium movement involved in cell motility (GO:0060294), and translation (GO:0006412) (Fig. 10E). For 4 A+, they comprised photosynthesis (GO:0015979), DNA-templated transcription (GO:0006351), maturation of 5.8 S rRNA from tricistronic rRNA transcript (GO:0000466), and maturation of LSU-rRNA from tricistronic rRNA transcript (GO:0000463) (Fig. 10F).

The cellular components for down-regulated proteins in CC-2931 included motile cilium (GO:0031514), chloroplast (GO:0009507), and axoneme (GO:0005930) (Fig. 10D). In CC-1009, the components were motile cilium (GO:0031514), chloroplast (GO:0009507), and axoneme (GO:0005930) (Fig. 10E). For 4 A+, the components consisted of chloroplast thylakoid membrane (GO:0009535), motile cilium (GO:0031514), and chloroplast (GO:0009507) (Fig. 10F).

Lastly, the molecular functions of down-regulated proteins in CC-2931 included ATP binding (GO:0005524), structural constituent of ribosome (GO:0003735), dynein intermediate chain binding (GO:0045505), dynein light intermediate chain binding (GO:0051959), metal ion binding (GO:0046872), microtubule binding (GO:0008017), microtubule motor activity (GO:0003777), minus-end-directed microtubule motor activity (GO:0008569), RNA binding (GO:0003723), and rRNA binding (GO:0019843) (Fig. 10D). In CC-1009, they comprised ATP binding (GO:0005524), DNA binding (GO:0003677), and structural constituent of ribosome (GO:0003735) (Fig. 10E). For 4 A+, they included ATP binding (GO:0005524), DNA binding (GO:0003677), GTP binding (GO:0005525), and GTPase activity (GO:0003924) (Fig. 10F).

**Fig. 11:**
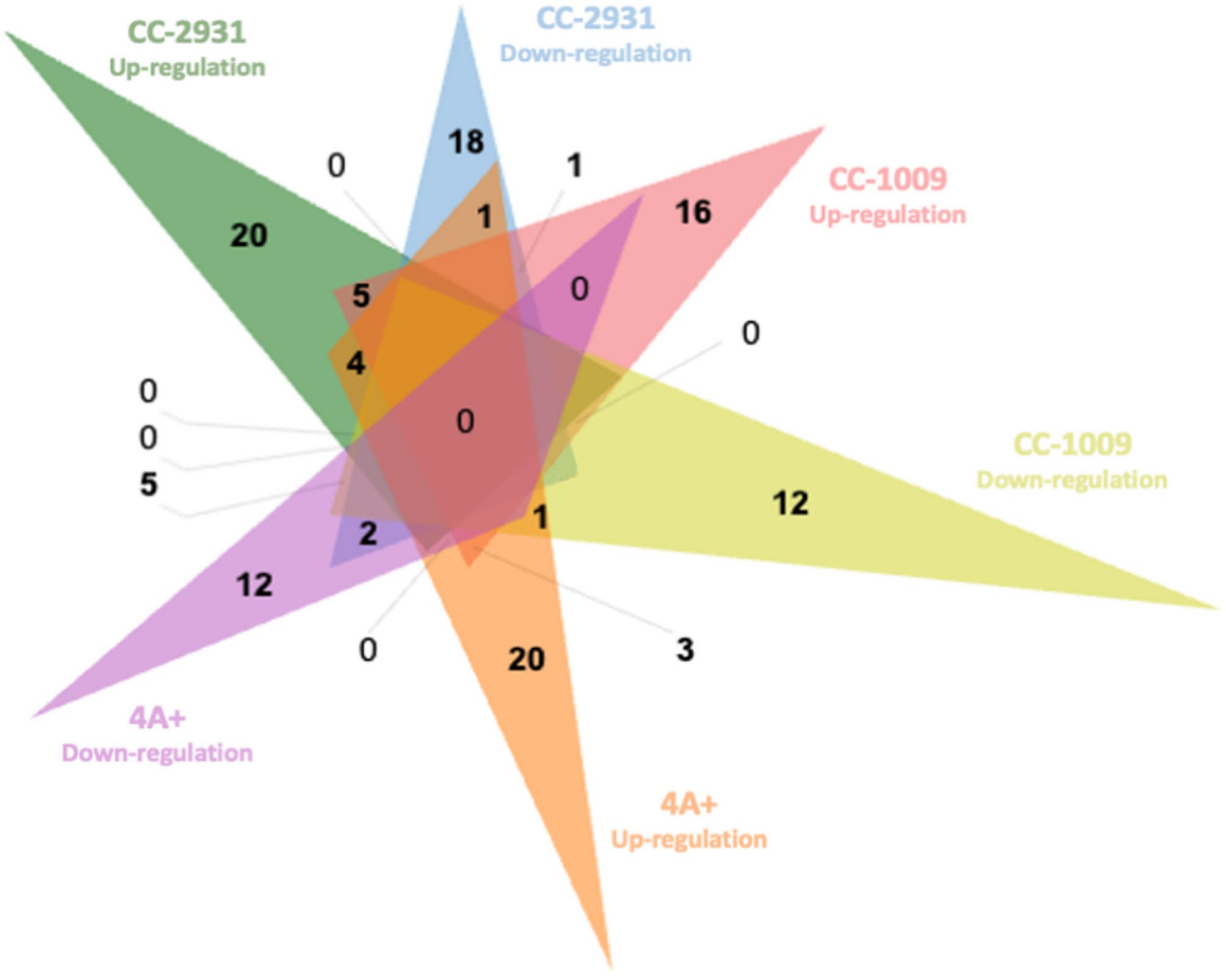
Venn diagrams illustrating the identified regulations in CC-2931, CC-1009, and 4 A+. The up-regulated protein is represented in green for CC-2931, pink for CC-1009, and orange for 4 A+. The down-regulated proteins are depicted in blue for CC-2931, yellow for CC-1009, and purple for 4 A+.

Protein data from CC-2931, CC-1009, and 4 A + were analyzed using a Venn diagram to illustrate their relationships (Fig. 11). For CC-2931 cells treated with norflurazon, twenty proteins and 18 proteins were exclusively upregulated and downregulated, respectively. For CC-1009, there were 16 proteins exclusively upregulated in this strain and 12 proteins that were exclusively downregulated. In the case of 4 A+, upregulated and downregulated proteins were 20 and 12, respectively. Five up-regulated proteins were shared between CC-2931 and CC-1009. The proteins found to be upregulated and downregulated, particularly in CC-2931 and CC-1009 were of interest. These proteins are listed in Table 1. Overall, these proteins corresponded to the GO terms found in each isolate.

**Table 1:** List of proteins showing up- and down-regulation in CC-2931, CC-1009, 4 A+, and up-regulation in both CC-2931 and CC-1009.

Uniprot ID	Function	Protein name	Peptide	*p*-value
CC-2931 up-regulation				
A8I4E9	Cell motility	Cilia- and flagella-associated protein 100 (Flagella-associated protein 100) (Modifier of inner arms 1 protein) (Mia1p)	KPDLNDPVLRAKLAK	0.016
P11093	Photosynthetic electron transport chain	Cytochrome b6-f complex subunit 4 (17 kDa polypeptide)	ADGVVLTEK	0.016
Q95AG0	Photosynthesis	Cytochrome f	AAPNLITPLR	0.043
A8HMZ4		Dynein regulatory complex subunit 5 (Flagellar-associated protein 155)	AAAGLDTLRLEDSDDDFK	0.031
P93107		Flagellar WD repeat-containing protein Pf20	AEKAAKK	0.043
P50565		Histone H2B.1 (H2B-I)	AAKMQEK	0.009
Q6RCE1	Cilium assembly	Intraflagellar transport protein 74 (Intraflagellar transport protein 71) (IFT-71) (Intraflagellar transport protein 72) (IFT74/72)	GKVMVICSNAK	0.030
P59774	Translation	Large ribosomal subunit protein bL36c (50 S ribosomal protein L36, chloroplastic)	CLNFAEMPTTPQYAMLPLCLIFLVCILAETKR	0.032
P11658		NADH-ubiquinone oxidoreductase chain 1 (EC 7.1.1.2) (NADH dehydrogenase subunit 1)	APYGSLEAAYK	0.002
P20113	ATP synthesis coupled electron transport	NADH-ubiquinone oxidoreductase chain 4 (EC 7.1.1.2) (NADH dehydrogenase subunit 4)	FLLSVFFPLLGGLVNTSPIARFLGHR	0.020
P08739	ATP synthesis coupled electron transport	NADH-ubiquinone oxidoreductase chain 5 (EC 7.1.1.2) (NADH dehydrogenase subunit 5)	AAGPARVPQQLR	0.012
Q39608	Nitrate assimilation	Nitrate transporter 2.1 (Nitrate assimilation related protein 3)	AGAKHVYGIECSTIAEQATQIVK	0.006
A8IEF3	Peptidyl-arginine methylation	Protein arginine N-methyltransferase 1 (EC 2.1.1.-)	KPDLNDPVLRAKLAK	0.039
A8I6P9	Intracellular protein transport	Protein transport protein Sect. 61 subunit beta	AGSQSQASTLVAR	0.002
A0A2K3DDJ2		Proteome of basal body protein 15	AALGPTAFARAGGFK	0.030
Q39593	Potassium ion transport	Putative sulfur deprivation response regulator	DSHFRSRYGAVVLR	0.016
A8JC00	tRNA splicing, via endonucleolytic cleavage and ligation	RNA-splicing ligase RtcB homolog (EC 6.5.1.8) (3’-phosphate/5’-hydroxy nucleic acid ligase)	AKGIAIRVASPK	0.006
P80028		Thioredoxin H-type (Trx-H) (Thioredoxin-CH1)	AAWDAQLAK	0.000
A8IC48	Protein urmylation	Ubiquitin-related modifier 1 homolog	DNMLRERPELFVK	0.005
P05724		Uncharacterized 14.4 kDa protein in 16 S rRNA region	CKTHQLVDLAK	0.024
CC-2931 down-regulation				
Q42689	Glutamine biosynthetic process	Glutamine synthetase, chloroplastic (EC 6.3.1.2) (GS2) (Glutamate–ammonia ligase)	AFALRGVTAK	0.021
P48267	Ribosomal small subunit assembly	Small ribosomal subunit protein uS7c (30 S ribosomal protein S7, chloroplastic)	AGAIQMVPR	0.011
A8IW34	‘De novo’ AMP biosynthetic process	Adenylosuccinate synthetase, chloroplastic (AMPSase) (AdSS) (EC 6.3.4.4) (IMP–aspartate ligase)	AHLLFDLHKEIDGLR	0.011
A8IB25	Ribosomal small subunit assembly	Small ribosomal subunit protein uS2 (40 S ribosomal protein SA)	AFEEPRLLILTDPR	0.017
P46870	Cell projection organization	Kinesin-like protein KLP1	AAAMATR	0.024
Q6UBQ3		Flagellar radial spoke protein 2	AAEALAATSAAEAVEELK	0.021
Q9SMH5	Establishment of protein localization to organelle	Cytoplasmic dynein 2 heavy chain 1 (Cytoplasmic dynein heavy chain 1b) (cDHC1b)	AAAGGSLIQPQQVFAEIK	0.003
P14273	Photosynthesis, light harvesting	Chlorophyll a-b binding protein of LHCII type I, chloroplastic (CAB) (LHCP)	AAAPKSSGVEFYGPNR	0.011
Q39586	Methionine biosynthetic process	5-methyltetrahydropteroyltriglutamate–homocysteine methyltransferase (EC 2.1.1.14) (Cobalamin-independent methionine synthase) (Methionine synthase, vitamin-B12 independent isozyme)	AAGIAVIGLDGTLYDQVLDTITWLGAIPPR	0.026
Q8HTL1	Translation	Large ribosomal subunit protein uL5c (50 S ribosomal protein L5, chloroplastic)	AIAGFKLRQQMPVGVTVTLR	0.016
A8JAM0		Dynein regulatory complex subunit 7 (Coiled-coil domain-containing protein lobo homolog) (Flagellar-associated protein 50)	ADGRLAALLN	0.019
Q9MBF8	Cilium movement involved in cell motility	Dynein-1-beta heavy chain, flagellar inner arm I1 complex (1-beta DHC) (Dynein-1, subspecies f)	AAEIKNGMDIFNIPQPQYK	0.023
A8HYJ1	Protein transport	Translocase of chloroplast 34 homolog, chloroplastic (Cr-TOC34) (TOC34p) (EC 3.6.5.-)	AEGRKQLTVLLLGK	0.023
A8IU92	Axoneme assembly	Cilia- and flagella-associated protein 20 (Basal body up-regulated protein 22) (Bug22p) (Flagellar-associated protein 20)	ASNYQSTTR	0.036
Q39610	Cell projection organization	Dynein alpha chain, flagellar outer arm (DHC alpha)	AAAFNDLYELDTSDPDEYK	0.010
P29683	Light-independent chlorophyll biosynthetic process	Light-independent protochlorophyllide reductase subunit N (DPOR subunit N) (LI-POR subunit N) (EC 1.3.7.7)	CGMIVYEIGIPYLDK	0.012
P49202	Translation	Small ribosomal subunit protein uS13 (40 S ribosomal protein S18)	AAGECSADELER	0.001
O20029	Translation	Small ribosomal subunit protein uS9c (30 S ribosomal protein S9, chloroplastic)	AILAKAVGRR	0.039
CC-1009 Up-regulation				
P36443		Uncharacterized 12.3 kDa protein in petA-petD intergenic region (ORF102)	ASKLRDFNAINK	0.00
P82678	Allantoin catabolic process	Allantoicase (EX 3.5.3.4) (Allantoate amidinohydrolase) (Protein EARLY ZYGOTE EXPRESSED 3)	AAISQQRTPLR	0.00
P46304	Cytochrome complex assembly	Cytochrome b6-f complex subunit 5 (Cytochrome b6-f complex subunit PetG) (Cytochrome b6-f complex subunit V)	RGDLATF	0.00
Q32063		Uncharacterized 6.2 kDa protein in psaC-petL intergenic region (ORF58)	ANFVLHSVK	0.03
P36492	Photosynthesis	Photosystem I P700 chlorophyll a apoprotein A2 (EC1.97.1.12) (PSI-B) (PsaB)	ATKLFPKFSQGLAQDPTTR	0.00
Q08684	Photosynthetic electron transport in photosystem II	Photosystem II CP43 reaction center protein (PSII 43 kDa protein) (Protein CP-43)	AAAAGFEK	0.01
P0DO19	Nonphotochemical quenching	Light-harvesting complex stress-related protein 3.1, chloroplastic (Chlorophyll a-b binding protein LHCSR3.1)	ASGLRQTPAR	0.00
A8HQD7	L-phehylalanine catabolic process	Phenylalanine 4-monooxygenase, chloroplastic (EC 1.14.16.1) (Aromatic amino acid hydroxylase) (Phenylalanine 4-hydroxylase)	ARWISSAPRPSTLVER	0.03
O20031	Photosystem I assembly	Photosystem I assembly protein Ycf3	AADYWKEAIR	0.03
P08197	Photosynthesis	Cytochrome c6, chloroplastic (Cytochrome c-552) (Cytochrome c-553) (Cytochrome c553) (Soluble cytochrome f)	AALEQYLDGGFK	0.01
P46305	Photosynthetic electron transport chain	Cytochrome b559 subunit beta (PSII reaction center subunit VI)	KSAEVITYPIFTVR	0.01
P04690	Microtubule-based process	Tubulin beta-1/beta-2 chain (Beta-tubulin)	AILMDLEPGTMDSVR	0.02
O63075		ATP synthase subunit a, chloroplastic (ATP synthase F0 sector subunit a) (F-ATPase subunit IV)	DLAKTQIGEEDYLK	0.01
A8JID5		Cilia- and flagella-associated protein 157 (Flagellar-associated protein 77)	AEQEHPQEGRPQQQEQGGEAR	0.02
Q42681		Histone H3 type 1	AARKTPATGGVK	0.01
P0CH11	Translation	Ubiquitin-ribosomal protein eL40z fusion protein	CYARLHPR	0.02
CC-1009 down-regulation				
Q5DM57		Intraflagellar transport protein 172	AAEANNPNTYIIR	0.023
A8JHB7	Glycine betaine biosynthetic process from choline	Fatty acid photodecarboxylase, chloroplastic (CrFAP) (EC 4.1.1.106)	AAAGPAGSEK	0.032
F8WQN2	Response to other organism	L-lactate oxidase (LOX) (EC 1.1.3.-) (Cr-LOX)	ADLSFLNLEEVEEEAK	0.041
Q8HTL7	DNA-templated transcription	DNA-directed RNA polymerase subunit beta C-terminal section (EC 2.7.7.6) (PEP) (Plastid-encoded RNA polymerase subunit beta C-terminal section) (RNA polymerase subunit beta C-terminal section)	ANVININILAR	0.006
Q7XJ96	Axonemal dynein complex assembly	Dynein regulatory complex subunit 4 (Growth arrest-specific protein 8 homolog) (Protein PF2)	AELRNKDR	0.013
A8IHT2		Dynein regulatory complex protein 11 (Flagellar-associated protein 82)	AILNPPPPSLASLLEDAAGDGKGKGK	0.004
Q6LCW8		Histone H3 type 2	AARKTPATGGVK	0.012
Q42694	Protein refolding	RuBisCO large subunit-binding protein subunit alpha, chloroplastic (60 kDa chaperonin subunit alpha) (CPN-60 alpha)	AADAKEIVFDQESR	0.015
Q39609	Nitrate assimilation	Nitrate transporter 2.2 (Nitrate assimilation related protein 4)	AKGNMWPVIK	0.007
P15451	Gibberellic acid homeostasis	Cytochrome c	ADLIAYLK	0.004
P54347		Histone H2B.4 (H2B-IV)	AAKEPKGDGEK	0.050
Q84U21	Response to antibiotic	Large ribosomal subunit protein uL22c (50 S ribosomal protein L22, chloroplastic)	ACEKIIKCLMSAAANAK	0.020
CC-2931 and CC-1009 up-regulation				
Q9XF62	Cell cycle	Microtubule nucleation factor SSNA1 (CrSSNA1) (13 kDa deflagellation-inducible protein)	AKITQELQILTK	0.03
A1JHN0		Homogentisate solanesyltransferase, chloroplastic (CrHST) (EX 2.5.1.117) (Homogentisate prenyltransferase)	DLCSSTGR	0.02
Q39580	Microtubule-based process	Dynein 8 kDa light chain, flagellar outer arm	ASGSSKAVIK	0.00
Q27YU7		Flagellar radial spoke protein (EC 1.-.-.-)	AAIQAGVPITCVQVEHSVLVR	0.02
P45841	Translation	Large ribosomal subunit protein eL31 (60 S ribosomal protein L31)	AVKEIRK	0.04
4 A + up-regulation				
Q94EY2	Protein ufmylation	Ubiquitin-fold modifier 1	FAAEEFK	0.003
P08681	Oxidative phosphorylation	Cytochrome c oxidase subunit 1 (EC 7.1.1.9) (Cytochrome c oxidase polypeptide I)	DIGLLYLVFAFFGGLLGTSLSMLIR	0.043
P17537	Photorespiration	Ribulose bisphosphate carboxylase small subunit, chloroplastic (RuBisCO small subunit)	AAPVAAPADAR	0.022
Q42684		Superoxide dismutase [Mn], mitochondrial (EC 1.15.1.1)	AAIEASFGSVDEMK	0.048
Q00471	Photosynthesis	Cytochrome b6	GGVGVGQATLTR	0.038
P32761	Intron homing	DNA endonuclease I-CeuI (EC 3.1.-.-) (23 S rRNA intron 1 protein)	FGLVVDPEFNVTQHVNGVK	0.001
P49644	Glucose metabolic process	Glyceraldehyde-3-phosphate dehydrogenase, cytosolic (EC 1.2.1.12)	AGIMLSPTFVK	0.015
A8JB22	Axonemal dynein complex assembly	Dynein regulatory complex subunit 2 (Coiled-coil domain-containing protein 65 homolog) (Flagellar-associated protein 250)	AAAATGAGLSVTGQGAGGGGQK	0.005
Q39584		Dynein 18 kDa light chain, flagellar outer arm	AFAMFDK	0.033
A0A2K3CNL6	Protein catabolic process	ATP-dependent Clp protease adapter protein CLPS1, chloroplastic	AASCLRPAPTASAQMMTR	0.015
P14217	Phenol-containing compound metabolic process	Arylsulfatase (AS) (EC 3.1.6.1) (Aryl-sulfate sulphohydrolase)	AKPAVRSR	0.011
P14149		Small ribosomal subunit protein uS12c (30 S ribosomal protein S12, chloroplastic)	DLPGVRYHIVR	0.023
A8JFU2		Cilia- and flagella-associated protein 65	AAAAADR	0.050
D2K6F1		Sodium/sulfate cotransporter 2 (SAC1-like transporter 2) (CrSLT2)	ADGSSVEASDYLYK	0.029
A8IYS6		Cilia- and flagella-associated protein 300 (Flagellar and basal body protein)	CVAVRYTKYYHK	0.043
Q08355		Ezy-1 protein	ACLADLRNRK	0.004
Q01656		Flagellar radial spoke protein 4	EAAKEAAPAAPAPER	0.015
P11471		Oxygen-evolving enhancer protein 2, chloroplastic (OEE2)	AALAGFAGAAALVSSSPANAAYGDSANVFGK	0.036
A8IVJ1		Protein Flattop homolog (Cilia- and flagella-associated protein 126) (Flagellum-associated protein 126)	AVEVPGTTER	0.011
Q8VXP3		Tbc2 translation factor, chloroplastic	AALLVHTFSGPLPGMAPGEVALSLWALGR	0.022
4 A + down-regulation				
P17746	Mitochondrial translational elongation	Elongation factor Tu, chloroplastic (EF-Tu)	EDQVDDKELLELVELEVR	0.021
A8IGK2		Elongation factor Ts, mitochondrial (EF-Ts) (EF-TsMt)	AFRVDSGAGGLVFPYVHQAAAPGLGK	0.039
Q39571	Protein transport	GTP-binding protein YPTC1	AFADEIGIPFLETSAK	0.042
Q9XHH2		Dynein axonemal light chain 1 (DNAL1) (Flagellar outer arm dynein light chain 1) (ODA-LC protein LC1)	ACKHLALSTNNIEK	0.024
Q7PCJ6	DNA-templated transcription	DNA-directed RNA polymerase subunit beta’’ (EC 2.7.7.6) (PEP) (Plastid-encoded RNA polymerase subunit beta’’) (RNA polymerase subunit beta’’)	AELEGLFYAK	0.010
P06541	Proton motive force-driven mitochondrial ATP synthesis	ATP synthase subunit beta, chloroplastic (EC 7.1.2.2) (ATP synthase F1 sector subunit beta) (F-ATPase subunit beta)	AHGGVSVFAGVGER	0.011
P14225	Photosynthesis	Photosystem I reaction center subunit psaK, chloroplastic (Light-harvesting complex I 8.4 kDa protein) (P37 protein) (PSI-K) (Photosystem I subunit X)	AARRSSVVVR	0.045
Q8RVC7		Sulfate permease 1, chloroplastic	ALGEFGSIVIVSSNFAFK	0.004
Q8HTL3	Ribosomal large subunit assembly	Large ribosomal subunit protein uL23c (50 S ribosomal protein L23, chloroplastic)	DRQYTFDVDLRLTKPQIK	0.025
P54345		Histone H2B.2 (H2B-II)	AAKEPKGDGEK	0.026
A8IR43	Maturation of 5.8 S rRNA from tricistronic rRNA transcript (SSU-rRNA, 5.8 S rRNA, LSU-rRNA)	Ribosome biogenesis protein WDR12 homolog	AEETQVLVKFVTKLPAHLR	0.020
P32974	Photosynthesis	Photosystem II reaction center protein L (PSII-L)	ARPNPNK	0.001

## Discussion

### Physiological responses

*Chlamydomonas reinhardtii* has served as the model organism for investigating the response to norflurazon, encompassing the identification of mutants exhibiting sensitivity or resistance to norflurazon and the modification of genes utilizing norflurazon as a selective marker^22,23,44^. Nonetheless, these investigations were conducted on a limited number of commonly used isolates, which exhibit considerable sensitivity to norflurazon. A study using four isolates—CC-3491, CC-503, CC-124, and CC-1010—indicated that a concentration of 1.5–3.5 µM in liquid culture resulted in complete cell mortality, whereas a concentration of 3–4 µM on plates also led to total cell death^22^. In another study on the development of a selective marker for genetic transformation, it was reported that the growth of the wild-type CC-1618 was completely inhibited by 0.5 µM of norflurazon^23^. Our work revealed that common *Chlamydomonas* laboratory isolates are very sensitive to norflurazon. Our research, using twenty wild-type isolates of *Chlamydomonas* covering most commonly used isolates and field isolates^45^demonstrated that roughly 50% of isolates tested were capable of growth on plates supplemented with 5 µM of norflurazon.

The two most resistant isolates identified in this study, CC-1009 and CC-2931, exhibited reduced growth compared to 4 A + in the absence of norflurazon. At a concentration of 5 µM norflurazon, both isolates grew significantly better than 4 A + at subsequent time intervals. The cell density at these time points was comparable to the density in the medium devoid of norflurazon, suggesting that this concentration did not influence the growth of these isolates. The effect of norflurazon on the cell viability of CC-2931 was significantly more apparent than that of 4 A+. As early as one day post-norflurazon treatment, the cell viability of CC-2931 decreased to 70–80%, implying the induction of mechanisms within the living cells that contribute to the high cell density observed in the growth curve. The abrupt decline in cell viability indicated a rapid reaction of CC-2931 to norflurazon. The F_v_/F_m_ value and cell color exhibited a small difference relative to the control. At 10 µM norflurazon, cell growth and photosynthetic efficiency significantly declined, and the cells exhibited a light green coloration, signifying damage to the photosynthetic system.

In the case of CC-1009, growth and cell viability were minimally affected by norflurazon, indicating the presence of mechanisms that effectively protect the cells against norflurazon toxicity. The mechanisms must particularly be protecting photosynthetic apparatus, as the F_v_/F_m_ values only marginally decreased at subsequent time points compared to CC-2931, which exhibited sensitivity to the presence of norflurazon in terms of F_v_/F_m_ values. This protection in photosynthesis then turns into cellular proliferation, cell viability, pigment accumulation and production, as well as the capacity to retain green coloration even after 96 h of exposure to 10 µM norflurazon.

Forward genetics aimed at enhancing carotenoid content through the screening of norflurazon-resistant strains was performed across various microalgal species, including *Synechococcus* PCC7942, *Haematococcus pluvialis*, *Chlamydomonas*, and *Chlorella zofingiensis*^27,46–49^. Numerous studies have employed forward genetics for strain enhancement; nevertheless, this approach may incur certain drawbacks. For example, the high-lutein mutant of C. *reinhardtii*, Mut-5, accumulated 3.0-fold more total chlorophyll and 3.6-fold more total carotenoids per cell compared to the parental strain, but exhibited reduced growth rates^50^. The two norflurazon-resistant isolates discovered in this work demonstrated only marginally diminished growth in the absence of norflurazon relative to the norflurazon-sensitive isolate. The carotenoid content in CC-1009 was comparable to that of other isolates; however, its chlorophyll content was significantly elevated. These findings imply that the ability of CC-1009 to tolerate a higher level of norflurazon, relative to other wild-type isolates, is not attributable to alterations in carotenoid biosynthetic genes. Instead, there is an enhancement of photosynthetic capacity and protection of the photosynthetic machinery.

Mutant and transgenic strains can tolerate concentrations of norflurazon up to 150 µM; however, the concentration necessary to effectively eliminate competition from other microalgae may not need to be excessively high. A study aimed at developing a selectable marker for the diatom *Phaeodactylum triconatum* revealed that this species exhibited 100% mortality at a concentration of 2.5 µM of norflurazon^51^. In the development of *Chlorella vulgaris* mutants for food applications, exposure to norflurazon at a concentration of 10 µM resulted in complete cell mortality^52^. The application of 0.02 µM norflurazon in conjunction with high light exposure resulted in a 50% reduction of carotenoids in *Haematococcus pluvialis*^53^. Therefore, low concentrations of norflurazon effectively suppress the growth of prevalent microalgae. The concentration of 5 µM did not adversely affect the growth and carotenoid production, as well as potentially other cellular products, of CC-1009 after 72 and 96 h. This indicates that this concentration may be suitable for large-scale algal production facilities in conjunction with wastewater remediation for the production of high-value compounds. Nevertheless, the TAP medium supplemented with acetate used in this investigation might not accurately represent the physiological dynamics of microalgal cells cultivated in ambient air or CO₂-enriched settings, which are more pertinent for treating agricultural wastewater. In order to evaluate the proteomic and physiological responses under realistic culture conditions for commercial use, more research is necessary. This limitation should be taken into account when interpreting the results.

### Proteomics responses

The protein responses at the subcellular level were examined in the three *C. reinhardtii* isolates. The most impacted GO terms across all isolates were related to photosynthesis, translation, and motility, indicating cellular alterations in the functional protein sets sensitive to norflurazon exposure. Alterations in the proteome due to norflurazon treatment have been documented in another common wild-type isolate, CC-125^54^, which was similar to 4 A + utilized in this study. Nine light-harvesting complex proteins of PSI were downregulated, indicating a decrease in photosynthetic activity. Pathways that were upregulated comprised chlorophyll production, cyclic electron transport, mitochondrial electron transport, and antioxidant processes. The proteins included glyceraldehyde-3-phosphate dehydrogenase, cytochrome c oxidase from the respiratory electron transport chain, thioredoxin, FSD1, and PRX1. It was concluded that norflurazon significantly disrupted the redox potential of cells, and the protective mechanism against mitochondrial oxidative stress and the essential requirement for ATP. Upregulation of these proteins suggests that an active maintenance of cellular redox balance and ATP generation is crucial for the synthesis of carotenoids and stabilization of photosynthetic machinery. This mechanism provides a plausible link between proteomic changes, carotenoid yield, and enhanced tolerance to norflurazon observed in our norflurazon-resistant isolates.

Similarly, our proteomic data revealed increased proteins associated with oxidative phosphorylation activities, including respiration and photosynthesis. Three proteins involved in photosynthesis are the RuBisCO small subunit, cytochrome b6, and chloroplastic oxygen-evolving enhancer protein. The antioxidative enzyme MnSOD was also increased. The down-regulated proteins associated with photosynthesis included ATP synthase, the photosystem I reaction center psaK, and the PSII reaction center PSII-L. Factors essential in translation, both in the nucleus and the mitochondria, were similarly downregulated, indicating a deceleration of protein synthesis. The differences in the specific proteins between the prior study and our research can be attributed to the significantly lower concentrations employed in the former, which were merely 0.1 and 1 µM, compared to 10 µM in our study. The duration of treatment varied, recorded as 6 h in their study versus 48 h in ours. At an earlier response at a far lower concentration, changes in the proteins involved in chlorophyll biosynthesis may have been more observable. The downregulation of light-harvesting complex protein may represent one of the initial responses to norflurazon, as it directly impacts the photosystem. At a longer time point in our study, the synthesis of the light-harvesting complex may not be as prominent as that of other photosynthetic proteins. Nonetheless, the primary pathways that were upregulated and downregulated in the presence of norflurazon were notably comparable.

Intriguingly, there were few proteins common to the two norflurazon-resistant isolates. No proteins were identified as overlapping proteins for downregulation, and only five proteins overlapped for upregulation, suggesting that the mechanisms conferring tolerance to norflurazon in these two isolates were distinct. These distinct proteomic profiles show that CC-1009 depends on upregulating protective and photosynthetic pathways, such as increased carotenoid and chlorophyll synthesis, to maintain the efficiency of photosynthesis. CC-2931, on the other hand, uses downregulating translation and motility-related processes as a survival strategy. Downregulation of these pathways might help conserve energy to increase survival. Understanding these contrasting strategies provides valuable insights for selecting isolates most appropriate for biotechnological applications.

The overlapping unregulated proteins were associated with the cell cycle, microtubule-based processes, and translation. Notably, a protein associated with prenyl production, homogentisate phytyltransferase (HPT), was increased in both isolates. This protein catalyzes the precursor synthesis of plastoquinone and tocopherol, indicating a significant and shared function of photosynthesis and photoprotection in norflurazon tolerance. Interestingly, other than its role as an electron acceptor in the photosynthetic electron transport chain, plastoquinone is also known as a cofactor for PDS enzyme^55,56^. Previous research demonstrated that norflurazon competes with NAD(P) and plastoquinone for binding to phytoene desaturase in the cyanobacterium *Synechococcus*^2^. In higher plants such as *Gentiana lutea* and *Oryza sativa*, norflurazon competes with plastoquinone for enzyme binding^2,57^. *Chlamydomonas* phytoene desaturase likely requires plastoquinone as a cofactor. We propose that the upregulation of ATP-producing pathways and HPT may lead to enhanced synthesis of NAD(P) and plastoquinone, which enable norflurazon resistance in CC-1009 and CC-1931. Further physiological and biochemical experiments will be required to support this idea.

Of the 16 elevated proteins in CC-1009, eight were associated with photosynthesis, comprising the cytochrome b_6_f complex, PsaB, CP43, LHCSR3, Ycf3, cytochrome 6, cytochrome b, and ATP synthase component. Furthermore, two uncharacterized chloroplastic proteins were also increased. This information confirmed that the survival of CC-1009 in the presence of norflurazon involved alterations in the expression of proteins mostly associated with photosynthesis and photoprotection. The role of proteins in photosynthesis is associated with the increased photosynthetic efficiency indicated by the F_v_/F_m_ value. LHCSR3 is a crucial protein implicated in non-photochemical quenching^58,59^. The action of norflurazon, by blocking carotenoid production, typically results in chlorophyll degradation and diminished chloroplast integrity. The increase of photosynthesis and photoprotection, particularly through non-photochemical quenching mediated by LHCSR3, enables this strain to preserve the integrity of the photosystem. Proteomic investigation of a high lutein-producing and norflurazon-resistant mutant of *C. reinhardtii*, designated Mut-5, also revealed LHCSR3 as a significantly upregulated and constitutively expressed protein^50^. Mut-5 exhibited reduced growth compared to the wild-type CC-125 under controlled conditions; nonetheless, PSII repair and NPQ appeared to be constitutively activated. Likewise, in our study, CC-1009 demonstrated reduced growth compared to 4 A+. Mut-5 also exhibited overexpression of several LHC proteins, serving as a reservoir for excess carotenoid production. In contrast, we observed no increase of LHC in CC-1009, as this strain did not acquire a significant quantity of carotenoid compared to Mut-5. The Mut-5 mutant exhibited downregulation in central metabolism, transcription, and translation. In CC-1009, the 12 downregulated proteins included one associated with transcription, specifically the plastid-encoded RNA polymerase subunit, and one related to translation, namely the large ribosomal subunit. The remaining proteins are implicated in flagella assembly and protein refolding, which do not fall within the same categories as Mut-5.

The response to norflurazon in CC-2931 involved an increase of proteins related to protein transport and modification. Proteins linked to photosynthesis, including chlorophyll a–b binding protein and light-independent protochlorophyllide reductase subunit N, were down-regulated. This finding corresponds to a significant drop in Fv/Fm value, indicating a decrease photosynthetic activity to mitigate oxidative stress. A substantial reduction in ribosomes and the synthesis of amino acids, specifically glutamine and methionine, was observed, indicating a decline in protein synthesis. This reduction correlates with the decreased growth observed at 48 h under 10 µM norflurazon. A considerable portion of downregulated proteins participated in cell motility. The proteins comprise kinesin-like protein, flagella radial spike protein, and three dynein proteins. The reduction in cell motility proteins indicated the reconfiguration of cellular proteins. A prior study indicated that reduced cell motility represents a prolonged adaptation to acidification in flagellated microalgae from various taxa, including *C. reinhardtii*^60^. The downregulation of protein synthesis, redistribution of proteins, and decreased photosynthesis and cell motility may represent the primary mechanisms by which CC-2931 survives in the presence of norflurazon.

## Conclusion

Agricultural wastewater contaminated with norflurazon can be utilized for the sustainable cultivation of microalgal biomass, facilitating the generation of high-value chemicals while concurrently remediating wastewater. Transgenic and mutant strains of the unicellular green alga *Chlamydomonas reinhardtii* have been generated in previous studies, but there is a concern regarding the environmental impacts on the release of strains that exhibit such high tolerance to herbicide. This study revealed that wild-type isolates of *C. reinhardtii* display differing degrees of tolerance to the herbicide norflurazon. Approximately 50% of the tested isolates exhibited resistance to 5 µM norflurazon concentration. CC-1009 did not show a growth defect or reduced carotenoid production at this concentration. The mechanisms enabling certain isolates to exhibit greater tolerance to norflurazon may vary. Proteomic results indicate that the activation of photosynthesis and photoprotective systems in CC-1009 is the primary cause of its resistance to norflurazon. In the case of CC-2931, the mechanisms involved included the deceleration of protein synthesis, photosynthesis, and cellular motility. More research will be needed to explore other mechanisms contributing to natural resistance to norflurazon. Additionally, future investigations should evaluate these isolates’ performance and proteomic profiles in ambient air and CO₂-enriched environments, which more accurately mimic large-scale wastewater treatments.

## Data Availability

Mass spectrometry proteomics data are available via ProteomeXchange: PXD057458 (https://proteomecentral.proteomexchange.org/cgi/GetDataset?ID=PXD057458) and JPOST: JPST003453 (https://repository.jpostdb.org/entry/JPST003453).
